# Delta coherence in resting-state EEG predicts the reduction in cigarette craving after hypnotic aversion suggestions

**DOI:** 10.1038/s41598-017-01373-4

**Published:** 2017-05-25

**Authors:** Xiaoming Li, Ru Ma, Liangjun Pang, Wanwan Lv, Yunlu Xie, Ying Chen, Pengyu Zhang, Jiawen Chen, Qichao Wu, Guanbao Cui, Peng Zhang, Yifeng Zhou, Xiaochu Zhang

**Affiliations:** 1 CAS Key Laboratory of Brain Function & Disease, and School of Life Sciences, University of Science and Technology of China, Hefei, Anhui Province China; 2 Department of Medical Psychology, Anhui Medical University, Hefei, Anhui China; 3 Anhui Mental Health Center, Hefei, Anhui China; 4 State Key Laboratory of Brain and Cognitive Science, Institute of Biophysics, Chinese Academy of Sciences, Hefei, Anhui China; 5 School of Humanities & Social Science, University of Science & Technology of China, Beijing, China; 6 Center for Biomedical Engineering, University of Science & Technology of China, Hefei, Anhui China

## Abstract

Cigarette craving is a key contributor of nicotine addiction. Hypnotic aversion suggestions have been used to help smoking cessation and reduce smoking relapse rates but its neural basis is poorly understood. This study investigated the underlying neural basis of hypnosis treatment for nicotine addiction with resting state Electroencephalograph (EEG) coherence as the measure. The sample consisted of 42 male smokers. Cigarette craving was measured by the Tobacco Craving Questionnaire. The 8-minute resting state EEG was recorded in baseline state and after hypnotic induction in the hypnotic state. Then a smoking disgust suggestion was performed. A significant increase in EEG coherence in delta and theta frequency, and significant decrease in alpha and beta frequency, between the baseline and the hypnotic state was found, which may reflect alterations in consciousness after hypnotic induction. More importantly, the delta coherence between the right frontal region and the left posterior region predicted cigarette craving reduction after hypnotic aversion suggestions. This suggests that the functional connectivity between these regions plays an important role in reducing cigarette cravings via hypnotic aversion suggestions. Thus, these brain regions may serve as an important target to treat nicotine addiction, such as stimulating these brain regions via repetitive transcranial magnetic stimulation.

## Introduction

In the last nationally representative survey in 2010 the prevalence of cigarette smoking for adult males in China was estimated to be almost 53%^1^. The need to understand effective smoking cessation strategies for this population is urgent. Craving, the subjective experience of a desire or intense need for substance use^2^ is commonly reported by people attempting to quit smoking. Thus, cigarette craving is an important target of smoking cessation treatments^3, 4^.

Hypnosis can be defined as an altered state of consciousness^5^. It consists of two components: hypnotic induction and hypnotic suggestion^6^. Hypnosis can help patients to lessen pain, and anxiety, and has even been used in the invasive clinical procedures accompanying interventional radiology and oncological surgery^7^. For smoking cessation, the data on the efficacy of clinical hypnosis are mixed^8^, with some studies reporting less than 25%^9^ and others reporting a high success rate of more than 80% abstinence^10^. In their recent Cochrane database review, Barnes *et al*.^11^ concluded that most studies had methodological flaws and any treatment success may be influenced by many factors. While not specifically mentioned craving, Oakley and Halligan^12^ note from a cognitive neuroscience perspective that the use of hypnotic induction and suggestion is a valid way to investigate brain activity in aberrant psychological states. However, to date the neural mechanisms underlying any effect of hypnotherapy on cigarette craving remain unclear.

The functional connectivity of different brain regions can be measured via Electroencephalography (EEG) coherence, which measures the synchrony of brain activity within or across different brain areas. Coherence is the stability of the EEG phase between sensor electrode pairs. It has been used to investigate brain changes associated with substance dependence^13–15^. For these reasons, this EEG technique is useful in investigate the neural basis of the effect of hypnotherapy on cigarette craving. Measuring EEG coherence while the brain is at rest is a feasible method^16^. With resting state connectivity measures researchers can study the flow of mental events, which happens within the mind or mind substitute of a conscious individual, in the absence of task performance^17^.

EEG coherence analysis has been employed in hypnosis research since the 1990’s^18^. Increased theta band functional connectivity in high susceptible participants was found as a result of hypnosis^19, 20^. High hypnotizable individuals also showed a reduction in upper alpha band coherence^21^. In the high susceptibility group, beta coherence decreased from baseline to hypnosis^19–21^.

The current study investigated EEG coherence changes between the baseline state and the hypnotic state in a resting-state condition. Given that nicotine abstinence causes increases in EEG power in low-frequency bands (delta and theta) and leads to reductions in high-frequency bands (alpha and beta) during a resting state^22^, we expected that the delta coherence and theta coherence would increase, and alpha coherence and beta coherence would decrease in the resting state as a result of hypnosis.

There is accumulating evidence that delta rhythm is associated with the activity of the brain’s reward circuit^23–25^. Moreover, the EEG changes in the frequency domain reflect drug craving^26^. Specifically, previous studies have found that delta power is related to subjective craving in smokers^27^. Comparable results were found in cocaine users in Reid *et al*.’s study^28^, who found that self-reported cocaine craving was correlated with delta power. If delta coherence will change in the resting state, we want to know whether the change is associated with cigarette craving after hypnosis. We hypothesized that resting-state delta coherence after the hypnotic induction would predict cigarette craving.

Hypnotic induction can alter activity in the default mode network^29^, which itself (such as the precuneus and posterior cingulated cortex) is positively related to craving and nicotine dependence and smoking cues^30–33^. However, the effects of hypnotic suggestions activate brain regions is associated with suggested events, e.g., visual hallucinations activate visual cortex^34^. Therefore, in this study we measured resting-state delta coherence after the hypnotic induction, but not after hypnotic suggestion. Though the brain activation we wanted to measure was not caused by smoking–related hypnotic suggestion, we could still make an assumption that the brain activation during resting state after hypnotic induction may predict the change of cigarette craving.

## Results

### Difference in EEG power between baseline and hypnotic states

EEG power increased significantly at bilateral frontal regions in delta band and at the right frontal area in theta band. In alpha, beta and gamma bands, EEG power decreased at both bilateral frontal and posterior areas (Table 1).

**Table 1:** EEG power (10 * log_10_(μV^2^/Hz)) in five difference frequency bands (hypnotic state > baseline). Note: *p < 0.05, **p < 0.01.

Regions	Baseline	Hypnotic state	*t*	*p*_*FDR*_
Delta				
LF	7.16 ± 1.91	8.08 ± 2.51	2.270	0.038*
RF	7.03 ± 1.99	7.93 ± 2.48	2.272	0.038*
LP	5.24 ± 2.16	6.00 ± 2.69	1.970	0.070
RP	5.49 ± 1.87	6.10 ± 2.92	1.519	0.151
Theta				
LF	0.57 ± 2.43	1.10 ± 2.39	1.920	0.072
RF	0.47 ± 2.62	1.13 ± 2.31	2.287	0.038*
LP	0.18 ± 3.14	0.62 ± 2.72	1.338	0.198
RP	0.28 ± 2.78	0.59 ± 3.01	0.926	0.361
Alpha				
LF	1.88 ± 3.79	0.40 ± 3.21	−3.457	0.003**
RF	1.68 ± 3.83	0.51 ± 3.06	−2.585	0.022*
LP	2.94 ± 4.12	1.21 ± 3.33	−3.890	0.001**
RP	3.46 ± 4.11	1.31 ± 3.55	−4.817	<0.001**
Beta				
LF	−6.22 ± 1.80	−7.20 ± 1.96	−3.822	0.001**
RF	−6.18 ± 1.95	−6.91 ± 2.16	−2.644	0.021*
LP	−6.31 ± 2.13	−7.53 ± 2.06	−4.727	<0.001**
RP	−6.04 ± 2.12	−7.59 ± 2.25	−5.969	<0.001**
Gamma				
LF	−11.15 ± 2.25	−12.60 ± 2.37	−3.244	0.005**
RF	−11.02 ± 2.22	−12.45 ± 2.70	−2.989	0.009**
LP	−12.59 ± 2.03	−14.05 ± 2.38	−4.240	<0.001**
RP	−12.21 ± 2.06	−14.00 ± 2.42	−4.919	<0.001**

### Difference between states in EEG coherence and TCQ

**Figure 1:**
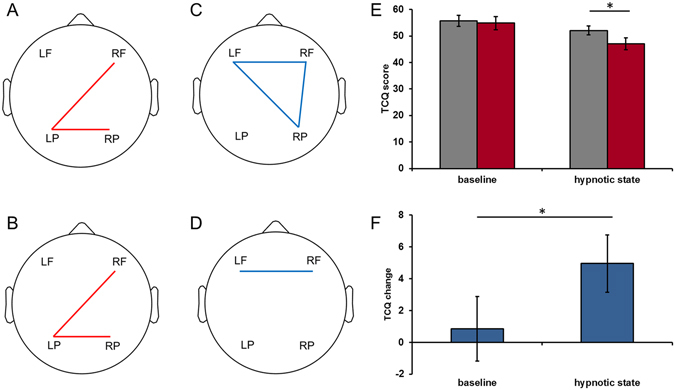
EEG coherence in four difference frequency bands and the TCQ score chang. (**A**) Delta coherence (RF-LP, LP-RP); (**B**) Theta coherence (RF-LP, LP-RP); (**C**) Alpha coherence (LF-RF, LF-RP, RF-RP); (**D**) Beta coherence (LF-RF); (**E**) The TCQ scores in the baseline and the hypnotic state; (**F**) The TCQ score change (TCQ scores measured before EEG scan – TCQ scores measured after EEG scan) in the baseline and the hypnotic state. Color coding: red (hypnotic state > baseline), blue (hypnotic state < baseline). TCQ: Tobacco Craving Questionnaire.

Four regions were defined as left-frontal (LF), right-frontal (RF), left-posterior (LP) and right-posterior (RP). There were significant increases in EEG coherence in delta (RF-LP, LP-RP) and theta (RF-LP, LP-RP) bands and significant decreases in alpha (LF-RF, LF-RP, RF-RP) and beta (LF-RF) frequency bands between the baseline and the hypnotic state (Table 2 and Fig. 1A–D).

**Table 2:** EEG coherence in five difference frequency bands (hypnotic state > baseline). Note: *p < 0.05, **p < 0.01.

Regions	Baseline	Hypnotic state	*t*	*p*_*FDR*_	Cohen’s *d*
Delta					
RF-LP	0.19 ± 0.06	0.22 ± 0.07	2.765	0.032*	0.46
LP-RP	0.09 ± 0.03	0.11 ± 0.04	3.461	0.010*	0.71
Theta					
RF-LP	0.24 ± 0.07	0.26 ± 0.06	3.060	0.017*	0.44
LP-RP	0.10 ± 0.03	0.11 ± 0.03	3.078	0.017*	0.53
Alpha					
LF-RF	0.39 ± 0.16	0.29 ± 0.15	−5.038	<0.001**	0.66
LF-RP	0.39 ± 0.09	0.35 ± 0.10	−3.065	0.017*	0.44
RF-RP	0.25 ± 0.09	0.20 ± 0.10	−4.402	0.001**	0.55
Beta					
LF-RF	0.14 ± 0.06	0.10 ± 0.03	−4.090	0.002**	0.79

The ANOVA analysis of the scores of Tobacco Craving Questionnaire-Short Form (TCQ^35^) revealed a significant main effect of states (*F* = 10.595, *p* = 0.002) while the main effect of testing time didn’t reach significance (*F* = 3.013, *p* = 0.090). Post-hoc comparison showed that TCQ scores before and after the EEG scan did not differ in the baseline (*t* = 0.417, *p* = 0.679). In contrast, the difference between TCQ scores in the hypnotic state reached statistical significance (*t* = 2.728, *p* = 0.010) (Fig. 1E). The interaction between states and testing time was significant (*F* = 4.486, *p* = 0.041) (Fig. 1F).

### Correlation results

**Figure 2:**
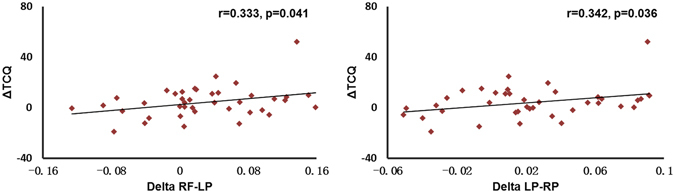
Delta coherence changes were correlated with TCQ change between states. (**A**) Delta coherence in the RF-LP; (**B**) Delta coherence in the LP-RP.

After control for the scores of Stanford hypnotic susceptibility scale (SHSS)^36^ and Fagerström Test for Nicotine Dependence (FTND)^37^, EEG coherence changes between states in delta band in RF-LP and LP-RP correlated with TCQ change between states (ΔTCQ) (*r* = 0.333, *p* = 0.041; *r* = 0.342, *p* = 0.036, respectively, see Fig. 2), while EEG coherence changes in other frequency bands and EEG power in all frequency bands were not significantly correlated with ΔTCQ (all *ps* > 0.05).

A stepwise linear regression analysis was performed with the delta coherence in RF-LP and LP-RP as independent variables, and ΔTCQ as dependent variable. ΔTCQ was independently predicted by the delta coherence in RF-LP only (*R* ^2^ = 0.104, *p* = 0.043).

## Discussion

The present study investigated EEG coherence changes between the baseline and the hypnotic state during a resting condition. In line with our hypotheses, a significant increase in EEG coherence in delta and theta frequency bands and significant decrease in alpha and beta frequency bands between the baseline and the hypnotic state. Previous studies found that nicotine abstinence causes increases in delta and theta power and leads to reductions in alpha and beta power during a resting state^22^. Further, we also found the same pattern in EEG power in the hypnotic state for smokers. That is to say, not only EEG power but also EEG coherence increased in delta and theta bands and decreased in alpha and beta bands.

Increases in theta power are correlated with drowsiness^38, 39^ and the transition from wakefulness to sleep^40^. Moreover, high delta and theta coherence is usually associated with lower levels of consciousness^41, 42^. Decreases in alpha frequency have been associated with slow reaction time^43^, diminished arousal and decreased vigilance^39, 44^. And the reduction in beta frequency may reflect the reduction of awareness^45^. Therefore, the increase in delta and theta coherence and decrease in alpha and beta coherence found in the present study may reflect alterations in consciousness after hypnotic induction. Several studies of EEG coherence during a resting hypnotized condition have been published recently^46–48^. They reported similar findings. For example, Fingelkurts *et al*.^47^ found an increase in the number of functional connections for theta frequency band and decrease of functional connectivity for beta frequency band during hypnosis. Therefore, we could speculate that increased low frequency and decreased high frequency may reflect consciousness/vigilance status. The higher the hypnotic depth of the smokers is, the more likely they are affected by the hypnotic aversion suggestions, and the more the level of cigarette cravings declines.

We found the TCQ scores decreased significantly following hypnosis, which demonstrates once again the effect of hypnotic aversion suggestions on reducing cigarette cravings. More importantly, the increase of delta coherence in RF-LP between the baseline and the hypnotic states during a resting condition can predicted the change in scores of cigarette cravings after hypnotic aversion suggestions. Previous studies demonstrate the link between delta power and cravings^23–25, 27, 28^. The delta power decrease may reflect increased activity of the dopaminergic brain reward system^25^, while increases in delta power reflect withdrawa^23, 24^. Our results further suggest that the delta coherence may also be associated with cigarette cravings. Specially, the delta coherence in RF-LP can predict the scores of cigarette cravings change between states, which suggests that the functional connectivity between the right frontal region and the left posterior region plays an important role in reducing cigarette cravings via hypnotic aversion suggestions. Frontal delta activity may reflect the state of craving. Littel *et al*.^27^ found that increases in craving were associated with left and right frontal increases in delta activity. Cocaine also produced a similar increase in delta coherence over the prefrontal cortex which was positively correlated with cue-induced cocaine craving^28, 49^. The left-posterior (CP5, CP1, P3, PO3, O1) may play a role in the development of cravings. Kim *et al*.^50^ found that alcohol craving results in the more complex EEG behavior in the left parietal (P3), and occipital regions (O1, O2). Although the EEG recording has a low spatial resolution, these findings in the present study support that the right frontal region and the left posterior region are activated during smoking-specific cue presentations. McClernon *et al*.^51^, using functional magnetic resonance imaging, found that cigarette craving is associated with the activation of the parietal, frontal, occipital, which provides further support for our point of view.

The present study has several clinical implications. First, while the results of the efficacy of clinical hypnosis for smoking cessation are mixed^8–10^, as pointed out by Barnes *et al*.^11^ there are many uncontrolled factors which influence the outcomes. So, it is difficult to predict the result of hypnotherapy for smoking cessation. The present study demonstrates that a measure of connectivity acquired from 8 min of resting-state EEG predicts subsequent cigarette craving. These data can be acquired rapidly, inexpensively, easily, and safely, therefore, the present EEG method has high potential clinical utility. Second, stimulating these brain regions via non-invasive brain stimulation, such as transcranial direct current stimulation or repetitive transcranial magnetic stimulation, may improve treatment efficacy^52^. Specially, by combining noninvasive treatments with hypnotherapy, it is possible to have an additive effect on treating the syndrome of addiction.

The present study has several limitations that should be considered. Firstly, regarding localization of coherence effects, the spatial resolution is limited because of low spatial resolution of EEG and the spatial averaging applied. Secondly, a self-reported tool to measure cigarette cravings change rather than an objective one was used in the current study, which can be biased by the influence of social desirability. Thirdly, in China, one distinctive smoking pattern in China is the disparity between the low prevalence among females and the high prevalence among males^53^. For this reason, only male smokers were recruited in the present study, which might limit the application of the current conclusions in clinic. Fourthly, although oscillation power and coherence are often adopted as two parallel measurement of brain activity, they may not be independent.

## Conclusion

The present study demonstrates that hypnosis causes increases in delta and theta coherence, and leads to reductions in both alpha and beta coherence, indicating alterations in consciousness after hypnotic induction. Moreover, the cigarette cravings after hynoptic suggestion were predicted by the functional connectivity between the right frontal region and the left posterior region during a resting condition. These areas can be target brain regions for future studies on cigarette craving and addiction.

## Materials and Methods

### Participants

A total of 44 male smokers took part in the study. Data from two participants were excluded because too much artifact. The final sample consisted of 42 smokers aged 20–48 (M = 26.0, SD = 5.9) with different hypnotic susceptibility measured by SHSS^36^ (M = 7.5, SD = 2.8). All of the participants smoked 8 or more cigarettes per day (M = 15.0, SD = 4.7) for a minimum of 2 years (M = 7.2, SD = 4.8). Their nicotine dependence was measured by FTND^37^ before experiment (M = 4.6, SD = 2.2). The participants provided written informed consent and the protocol was approved by the Human Ethics Committee of University of Science and Technology of China. The experiments were performed in accordance with the approved protocol.

### Experimental Design

After the preparation of EEG recording, 8-minute resting state EEG was recorded in baseline and just after hypnotic induction in hypnotic state with participants comfortably seated and eyes closed. After that, a smoking disgust suggestion was performed. For example, smokers were told that cigarettes would smell and taste like excrement (according to Spiegel *et al*.^54^). We measured resting EEG before smoking disgust suggestion because we wanted to find an EEG measurement to predict the effect of hypnosis rather than directly show the effect of smoking disgust suggestion.

**Figure 3:**
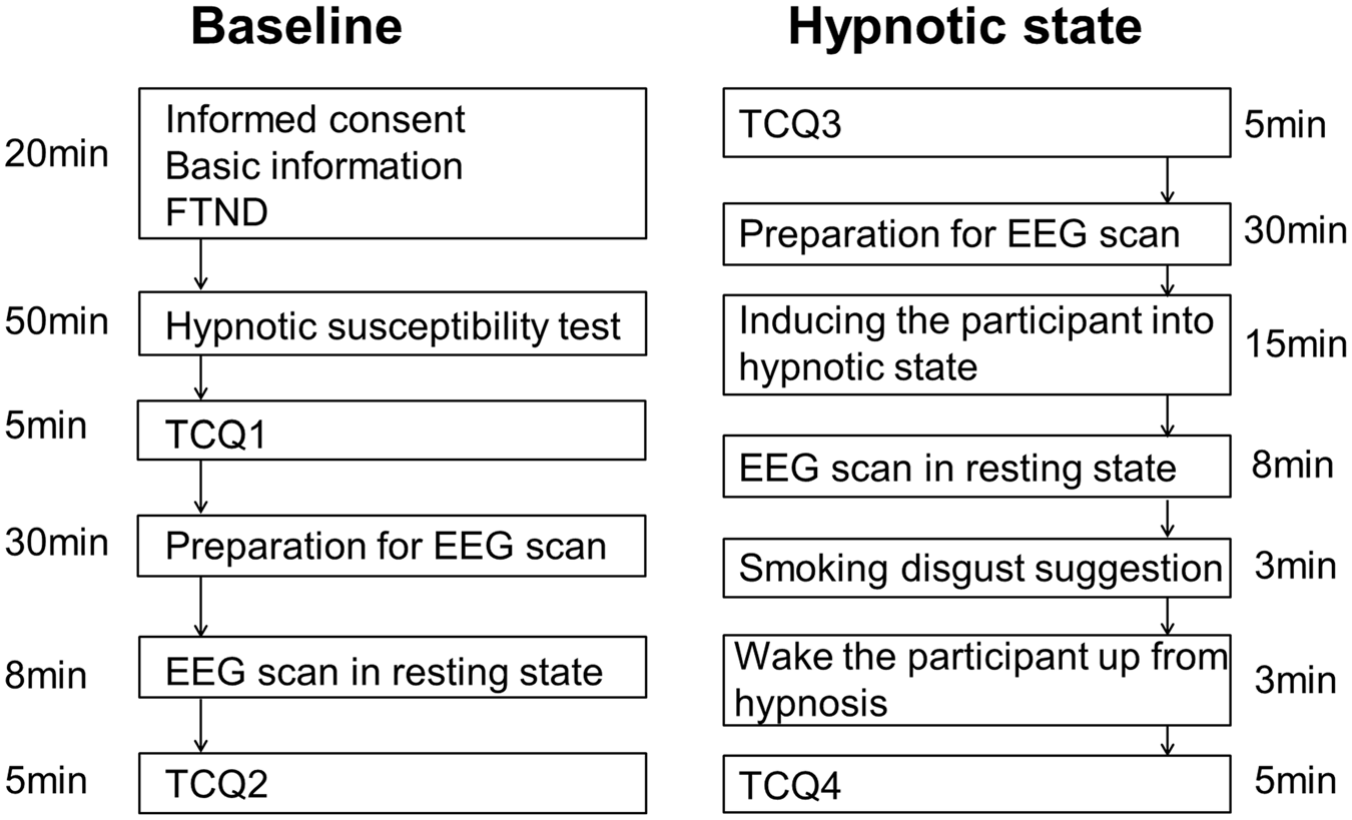
The flow chart of the experiment. FTND: Fagerström Test for Nicotine Dependence; TCQ: Tobacco Craving Questionnaire.

The experiment in the baseline was always implemented before the experiment in the hypnotic state, to provide a baseline measure of brain activity before assessment of any effect of the hypnotic disgust suggestion. Cigarette craving was assessed before and after the EEG recording in each state by TCQ^35^. The procedure is shown in Fig. 3.

### EEG recording

A SynAmps 2 amplifier (NeuroScan, Charlotte, NC, U.S.) was used for EEG recording. A 64-channel electrode cap with Ag/AgCl electrodes placed according to the extended international 10–20 system was laid on the scalp. The reference electrode was located on the tip of participant’s nose while the ground electrode was AFz. Electrical activities at both left and right mastoids were recorded. Vertical electrooculogram (EOG) was recorded with a bipolar-channel located above and below the left eye, and horizontal EOG was recorded with a bipolar-channel placed by the side of the outer canthus of each eye. Impedance between the reference electrode and each recording electrode was kept under 5 kΩ. 0.05–100 Hz filtered alternating current signals were continually recorded and digitized by 24 bit resolution with a sampling rate of 500 Hz.

### EEG data processing

EEG data processing was performed using MatLab (MathWorks Inc., Natick, MA, USA), EEGLab^55^ (http://sccn.ucsd.edu/eeglab/) and customized MatLab Code. Four electrodes (M1, M2, CB1, CB2) were excluded from analysis. EOG, EMG and other artifact was rejected manually and using ICA methods. We removed 1.77 ± 2.00 components from total 62 ICA components in this study. Correcting artifacts using ICA method is widely used in EEG studies, some of which calculated coherence or other measurement relevant with phase^56–60^. We compared coherence after visual artifact rejection only and after additional ICA component rejection to investigate whether there was significant differences between the two approaches. We performed a 2 (approaches) × 2 (states) × 5 (frequency bands) × 6 (coherence in region pairs) repeated measure ANOVA and found that there was no significant difference between the two approaches (*F* = 0.036, *p* = 0.851).

The EEG of first second and data beyond 5 minutes was excluded from analysis. EEG data was segmented into epochs with 512 points (1024 ms) and detrended. Epoches contained values exceeding ±100 μV were rejected. Remain epoches were re-referenced to average reference.

Four regions were defined as left-frontal (LF: FP1, AF3, F7, F3, FC5), right-frontal (RF: FP2, AF4, F8, F4, FC6), left-posterior (LP: CP5, CP1, P3, PO3, O1) and right-posterior (RP: CP6, CP2, P4, PO4, O2). EEG power spectrum was calculated using FFT with a Hamming window (512 points) without overlap and averaged in each region in delta (0.1–3.9 Hz), theta (4–7.9 Hz), alpha (8–12.9 Hz), beta (13–29.9 Hz) and gamma (30–49.9 Hz) frequency band^61^.

The average cross spectrum of two electrodes, calculated from the complex conjugate of Fourier coefficients, was squared and normalized by the average residual power spectrum of each region to calculate the coherence between two electrodes across epochs at each frequency^62, 63^. All distances between two electrodes used in our study were larger than 5 cm, which is the estimated spatial resolution of EEG^64–66^, artifacts from volume conduction should not be an issue. And because Laplacian derivation might diminish discrimination at lower frequences and make coherence erroneous^67, 68^, we didn’t adopt surface Laplacian method in this study. Coherence in delta, theta, alpha, beta and gamma frequency band was calculated by averaging coherence values in each frequency interval. Frequency band coherence across multiple electrode combinations was averaged to calculate connectivity between regions.

### Statistic analysis

Statistic analysis was performed using IBM SPSS Statistics 20.0 (IBM Corp, Armonk, NY, USA) and JMP 10.0.0 (SAS Institute Inc, Cary, North Carolina, USA). A 2 (states) × 2 (testing time: before/after EEG scan) repeated measure ANOVA was performed to TCQ scores and post-hoc comparisons was implemented. TCQ change between states was calculated according to the following equation: ΔTCQ = (TCQ3 − TCQ4) − (TCQ1 − TCQ2). The difference of EEG power and EEG coherence between the baseline and the hypnotic state was tested by two-tailed paired t-test. Multiple comparisons were corrected by false discovery rate (FDR) method. The correlation between ΔTCQ and difference of power and coherence between states (hypnotic state > baseline) that reached significance level was tested by two-tailed partial correlation, with the SHSS scores and the FTND scores controlled. Data controlled the SHSS and the FTND scores were calculated using the least square method. Stepwise linear regression analysis was performed to detect whether the EEG coherence change predicted the ΔTCQ.
